# Visual impairment in the hearing impaired students

**DOI:** 10.4103/0301-4738.57155

**Published:** 2009

**Authors:** Parikshit Gogate, Nikhil Rishikeshi, Reshma Mehata, Satish Ranade, Jitesh Kharat, Madan Deshpande

**Affiliations:** 1 Department of Community Eye Care, H.V. Desai Eye Hospital, Pune, India; 2 Department of Pediatric Ophthalmology, H.V. Desai Eye Hospital, Pune, India

**Keywords:** Deaf and mute, hearing-impaired children, vision-impaired, refractive errors

## Abstract

**Background:**

Ocular problems are more common in children with hearing problems than in normal children. Neglected visual impairment could aggravate educational and social disability.

**Aim:**

To detect and treat visual impairment, if any, in hearing-impaired children.

**Setting and Design:**

Observational, clinical case series of hearing-impaired children in schools providing special education.

**Materials and Methods:**

Hearing-impaired children in selected schools underwent detailed visual acuity testing, refraction, external ocular examination and fundoscopy. Ocular motility testing was also performed. Teachers were sensitized and trained to help in the assessment of visual acuity using Snellen's E charts. Refractive errors and squint were treated as per standard practice.

**Statistical Analysis:**

Excel software was used for data entry and SSPS for analysis.

**Results:**

The study involved 901 hearing-impaired students between four and 21 years of age, from 14 special education schools. A quarter of them (216/901, 24%) had ocular problems. Refractive errors were the most common morbidity 167(18.5%), but only 10 children were using appropriate spectacle correction at presentation. Fifty children had visual acuity less than 20/80 at presentation; after providing refractive correction, this number reduced to three children, all of whom were provided low-vision aids. Other common conditions included strabismus in 12 (1.3%) children, and retinal pigmentary dystrophy in five (0.6%) children.

**Conclusion:**

Ocular problems are common in hearing-impaired children. Screening for ocular problems should be made mandatory in hearing-impaired children, as they use their visual sense to compensate for the poor auditory sense.

Available evidence suggests that ophthalmologic screening and detection of visual problems in deaf children is important because the vast majority of knowledge is obtained through the senses of sight and hearing, some through the tactile, kinesthetic and olfactory senses. When one of these is seriously impaired, the other is used to compensate the disability, so the deaf population may compensate by making greater use of visual-perceptual cues than their normal hearing peers, and thus even a mild refractive error may reduce the visual cues available to the deaf mute person.[1]

Secondly, many researches have reported high incidences of ophthalmologic abnormalities among deaf children compared with the hearing population of the same age.[2,3] A review of the literature suggests 35 to 57% visual defects among hearing-impaired children.[2–8] Therefore, particular attention must be paid to ocular abnormalities in deaf children, as their early detection and proper treatment are the best assurances for the maximum possible social and professional performance of these students.[1]

The aim of the study was to determine the nature and prevalence of ophthalmologic abnormalities in students attending deaf mute schools (special education schools) in a large city in western India.

## Materials and Methods

Permission was sought and obtained from the principals of special education schools for the hearing impaired in and around Pune city. All these schools had admission policies to include only hearing-impaired children; normal children and those with other handicaps were officially not admitted into the schools. A team of ophthalmologists, optometrist and social worker visited each school. The principals and teaching staff of the schools were briefed about the eye examination.

Each child's hearing and speaking ability was recorded as reported by the teachers, along with the cause and type of deafness, where this information was available. The hearing ability had been measured in decibels. Hearing impairment was classified as mild, moderate, severe and profound deafness as per World Health Organization norms.

The children were examined with a school teacher near them and they responded by sign language that was interpreted by the teacher. The ophthalmologic work-up included visual acuity assessment, pupillary evaluation, ocular motility examination, and alternate cover test and fundus examination.

Snellen's E–Chart was used for examining children over seven years of age, and Kay picture chart was used for younger children. Near-vision testing (33 cm) was done first, and then visual acuity at six meters was examined. The child was required to correctly match the direction of his fingers to the arms of the E. After the child responded easily, each eye was tested separately. Cycloplegic refraction was done where indicated. Myopia was defined as an error more than or equal to −0.5 diopter (D), hypermetropia as more than or equal to +1.0 D, and astigmatism as more than or equal to + 0.5 D. Amblyopia was defined as best corrected visual acuity of less than 20/200 in either eye resulting from anisometropia, strabismus or large astigmatic error. Extraocular muscle imbalance was noted when eye misalignment exceeded 10 prism diopter (>5 degrees).

Children who needed more detailed evaluation were referred to the H. V. Desai Eye Hospital's pediatric ophthalmology department.

## Results

Nine hundred and one students of 14 schools for the hearing impaired in and around Pune city were examined. Males comprised 554/901 (61.5%), and the age ranged from four to 21 years, averaging 12.7 years (SD 4.35). Hearing impairment had been certified by the doctor before admission to the school.

In this study six hundred and eighty-five children 685/901 (76.0%) had a normal ophthalmologic examination, while 216/901 (24.0%) children had ocular problems. The details of ocular morbidity observed are shown in Table 1.

**Table 1:** Ocular problems found in hearing-impaired students

Treatment given	No. of children	Percentage
Normal	685	76.0
Refractive errors	167	18.5
Conjunctivitis	06	0.5
Dacryocystitis	02	0.2
Dry Eye	04	0.4
Blepharitis	01	0.1
Chalazion	01	0.1
Hordeolum externlume	01	0.1
Heterochromia iridis	1	0.1
Night Blindness	2	0.2
Squint	12	1.3
Retina	10	1.1
Vitamin A Deficiency	1	0.1
Cornea	1	0.1
Ptosis	1	0.1
Others	5	0.6
Glaucoma	1	0.1
Total	901	100.0

In this study, 257/901 (28.5%) children were profoundly deaf, 476/901 (52.8%) were severely deaf, 94 (10.4%) were moderately hearing-impaired and 74/901(8.2%) children were mildly deaf. The cause of deafness was known in 194/901(21.5%) children. The majority, 382/901 (42.4%) were not able to speak at all, 369/901 (41.0%) were able to speak, and only 150/901(16.6%) children were verbally articulate.

Prevalence of refractive errors in the present study was 167/901 (18.5%). Hypermetropia was found in 41/167 (24.5%) children, while myopia was found in 113/167 (67.7%) and astigmatism was found in 13/167 (7.8%) children. Of these, ten were wearing spectacles at the time of examination, and 18 children with refractive errors had vision better than 20/40 before they were checked. With appropriate spectacle correction, 104 had best corrected visual acuity better than or equal to 20/30. Visual acuity less than 20/60 on presentation was detected in 50 (5.5%) children. All except three (0.3%) improved to better than or equal to 20/60. These three children were provided low-vision aids.

Most children had never undergone any eye checkup previously; 232/901 (25.7%) children had been examined the previous year by our team, though no spectacles were dispensed to the children and only a prescription for glasses had been given at that time.

## Discussion

Hearing-impaired children rely almost entirely on their visual senses to learn about their environment. If a visual handicap is added to the auditory handicap it would affect such a child more than it would affect a normal child. Refractive errors and amblyopia are easily treatable and it would be a shame if such a hearing-impaired child does not get proper eye care attention. Our study showed that 24.0% hearing-impaired children had eye problems as compared to 48 (43.6%) ophthalmic abnormalities in sensorineural deaf children in the UK,[2] 100 (35.8%) with ocular problems in deaf mute students in China,[6] 178 (33%) having minor ocular abnormalities in Australia[7] and 95/165 (57.6%) deaf mute children having ocular abnormalities in Malaysia.[4] Table 2 compares the findings of this study with other similar studies in published literature. The percentage of ocular problems was more in some other studies as they may have considered children with severe hearing loss and perhaps additional handicap. An evidence-based review of ophthalmic disorders in deaf children done in Greece found that the overall quantity of evidence in the literature concerning deaf children and their ophthalmic problems was low.[9] The prevalence of ocular problems in deaf mute children was high and may remain undetected for years, having a serious impact on the development of their communication skills. Only 10 of the 167 children with refractive errors were using spectacles. Screening for ophthalmic problems amongst the deaf should be encouraged and their caretakers (parents and teachers) should be sensitized to the same.

**Table 2:** Comparison of ocular problems in hearing-impaired children

Name	Country	Year	Children examined	Ocular Problem	Refractive Error	Usher's	Fundus	Motility
Guy *et al*.[2]	United Kingdom	2003	122	110/122	43	6/122		
				(90.1)	(39.1)	(4.9)		
Hanioğlu-Kargi Se[3]	Turkey	2003	104	42/104	31/104		9/104	
				(40.4)	(29.3)		(8.6)	
Elango *et al*.[4]	Malaysia	1994	165	95/165	23		35.2 Rubella	
				(57.6)	(13.9)			
Siatkowski, *et al*.[5]	USA	1994	54	33/54	24/54		3/54	2/54
				(61.1)	(44.4)		(5.5)	(3.7)
Ma *et al*.[6]	China	1989	279	100/279	50/279	2/279	80/279	
				(35.8)	(17.9)	(0.7)	(28.6)	
Nicol *et al*.[7]	Australia	1988	78	26/78				
				(33)				
Regenbogen *et al*.[8]		1985	150	68				12
				(45.3)				(1.3
Present study	India	2008	901	216/901	167/901	5/901	10/901	12/901
				(24.0)	(18.5)	(0.6)	(1.1)	(1.3)

Figures in parentheses indicate percentage

Eye care professionals administering visual acuity tests should be sensitive to the communication needs of the deaf children. A comprehensive eye care examination is mandatory and should be repeated every few years as the child's visual and refractive status may change. Ophthalmologists should especially look out for retinal pigment abnormalities.

In the study group, the most common ocular abnormality was refractive error. Of the 901 deaf children examined, 167/901 (18.5%) had one or more refractive errors. The frequency of refractive errors in the present study was twice that found in the normal hearing population.[10,11] The next most common ocular abnormality was found to be strabismus, which has been determined at different rates in previous studies. The incidence of manifest strabismus has been cited as 1.3% and 3.7% from overseas studies.[8,5] In our study, 12/901 (1.3%) of the children had strabismus, which was significantly greater than in the normal population. Higher prevalence of refractive and strabismic errors in the hearing-impaired population, who may be amenable to spectacle, surgical or orthoptic treatment, makes early diagnosis essential because this population is especially dependent upon vision for their maximal cognitive, psychological and emotional development.

The present study shows an increased prevalence of refractive errors and other ocular problems in deaf mute children.

Ophthalmologists play an important role in organizing such screening programs so that related diseases may be diagnosed and treated. Early ophthalmologic assessment of the hearing-impaired is advisable to detect any ocular visual impairment, followed by correction to help in the academic and social performance of these children.
